# A prospective study of pre-trauma risk factors for post-traumatic stress disorder and depression

**DOI:** 10.1017/S0033291716000532

**Published:** 2016-06-28

**Authors:** J. Wild, K. V. Smith, E. Thompson, F. Béar, M. J. J. Lommen, A. Ehlers

**Affiliations:** 1 Department of Experimental Psychology, University of Oxford, Oxford, UK; 2 King's College London, London, UK

**Keywords:** Depression, paramedics, post-traumatic stress disorder, resilience, risk

## Abstract

**Background:**

It is unclear which potentially modifiable risk factors best predict post-trauma psychiatric disorders. We aimed to identify pre-trauma risk factors for post-traumatic stress disorder (PTSD) or major depression (MD) that could be targeted with resilience interventions.

**Method:**

Newly recruited paramedics (*n* = 453) were assessed for history of mental disorders with structured clinical interviews within the first week of their paramedic training and completed self-report measures to assess hypothesized predictors. Participants were assessed every 4 months for 2 years to identify any episodes of PTSD and MD; 386 paramedics (85.2%) participated in the follow-up interviews.

**Results:**

In all, 32 participants (8.3%) developed an episode of PTSD and 41 (10.6%) an episode of MD during follow-up. In all but nine cases (2.3%), episodes had remitted by the next assessment 4 months later. At 2 years, those with episodes of PTSD or MD during follow-up reported more days off work, poorer sleep, poorer quality of life, greater burn-out; and greater weight-gain for those with PTSD. In line with theories of PTSD and depression, analyses controlling for psychiatric and trauma history identified several pre-trauma predictors (cognitive styles, coping styles and psychological traits). Logistic regressions showed that rumination about memories of stressful events at the start of training uniquely predicted an episode of PTSD. Perceived resilience uniquely predicted an episode of MD.

**Conclusions:**

Participants at risk of developing episodes of PTSD or depression could be identified within the first week of paramedic training. Cognitive predictors of episodes of PTSD and MD are promising targets for resilience interventions.

## Introduction

Emergency workers are routinely exposed to potentially traumatic events and are at risk of developing stress-related mental disorders. Whilst post-traumatic stress disorder (PTSD) is the more common outcome following trauma, evidence suggests that major depression (MD) can develop without co-occurring PTSD in the aftermath of trauma and is a common outcome following repeated exposure to stressful life events (Wang *et al.* 2008). There is accumulating evidence linking PTSD and MD to risk of developing cardiovascular disease (Joynt *et al.* 2003; Kubzansky *et al.* 2007), diabetes and Alzheimer's disease (Yaffe *et al.* 2010) and even early death (Xue *et al.* 2012). To prevent negative effects of repeated trauma exposure on emergency workers’ mental and physical health, there is a need to identify risk factors for PTSD and MD that can be targeted with resilience interventions. Some predictors, such as psychiatric history, are fixed and cannot be modified. However, others such as cognitive styles and behavioural traits are modifiable by training to reduce the risk of developing PTSD and MD, and their negative impact on health. Theories of PTSD and MD and empirical studies suggest several potentially modifiable predictors for both PTSD and MD. These include cognitive styles (e.g. attributional style; Ehring *et al.* 2008), maladaptive post-traumatic cognitions (Abramson *et al.* 1999), cognitive responses to memories of negative events (e.g. rumination; Murray *et al.* 2002), coping styles (Clohessy & Ehlers, 1999), psychological traits (e.g. neuroticism; Engelhard & van den Hout, 2007) and poor social support (Ozer *et al.* 2003).

To date, most studies investigating risk factors for PTSD and depression linked to traumatic events have assessed individuals after trauma, making it difficult to identify vulnerability factors that pre-date an individual's exposure to trauma. The exception is prospective studies of police officers (Wang *et al.* 2008), firefighters (Bryant & Guthrie, 2005; Heinrichs *et al.* 2005) and military personnel (Wittchen *et al.* 2013; Lommen *et al.* 2014). A number of studies have found evidence for associations between pre-trauma cognitions (Bryant & Gurthrie, 2005), pre-trauma trait dissociation (Nash *et al.* 2015), self-efficacy (Heinrichs *et al.* 2005), and trait anger (Lommen *et al.* 2014) and subsequent PTSD. Lower self-worth during police academy training has been linked to later depression (Wang *et al.* 2008).

However, the evidence on pre-trauma risk factors is still sparse and most studies have been limited by short follow-ups, samples of mostly male participants or methodologies that rely on assessing symptom severity once during follow-up rather than regularly. Assessing severity at one follow-up time point may limit the conclusiveness of the findings since both PTSD and MD have a high rate of spontaneous recovery, and episodes of high symptoms may be missed if the traumatic event occurred soon after baseline and participants recovered by follow-up. It is therefore desirable to monitor participants regularly and document any episodes of PTSD or depression that occur during follow-up. The present study took this approach to investigate episodes of PTSD or MD in a large sample of newly recruited paramedics. The study had three aims: (i) to investigate prospectively whether new recruits undergoing paramedic training are at risk of developing episodes of PTSD or MD; (ii) to investigate whether episodes of PTSD or MD predict poorer well-being at 2 years; (iii) and to identify variables that can be assessed before exposure to trauma that predict who will develop an episode of PTSD or MD, and are possible targets for resilience training.

## Method

Ethical approval was granted by the National Health Service's Lewisham Local Research Ethics Committee.

### Participants

Participants were new recruits to the London Ambulance Service (LAS), London, UK, who were recruited over a 12-month period in a government-based initiative to increase the workforce of emergency medical technicians by the 2012 Olympics held in London and attended one of several paramedic training courses held at a UK paramedic training centre in London. The training courses were part of a 3-year paramedic training programme, which consisted of 6 weeks of classroom-based learning at the start of the programme followed by almost 3 years working as a trainee paramedic, attending call-outs with a qualified paramedic where the trainees were regularly exposed to potentially traumatic incidents. During the 3-year programme, participants also completed a number of essays and examinations. They received information on the effects of stress on well-being and no specific training on how to be mentally resilient. Researchers approached all attendees of each new training course at the start of their paramedic training course, and presented information about the study, including giving out the Participant Information Sheet. Interested participants provided their email addresses. Researchers then emailed interested participants to set up an assessment appointment the same week and to make available again the Participant Information Sheet. Participants were encouraged to ask questions about the study by email and in person. At the assessment appointment, participants were asked if they had any questions and written consent was obtained.

Of the 472 student paramedics approached, 456 (96.6%) agreed to participate. Three withdrew before completing baseline assessments, and 453 participants completed baseline assessments. Of these, 67 (14.8%) dropped out of the study during the 2-year follow-up. The most common reason for leaving the study was that the student had left the LAS because they had failed their examinations (*n* = 29) or left the course for another reason (*n* = 33). Five participants continued to work at the LAS, but attended fewer than two follow-up assessments and were excluded from the analysis. Table 1 shows participant characteristics at baseline.

**Table 1.** Demographic and baseline measures of participants assessed within 1 week of starting paramedic training

Variable		Sample (*n* = 453)
Mean age, years (s.d.)		30.31 (7.69)
Gender, *n* (%)	Men	264 (58.3)
	Women	189 (41.7)
Mean duration of education, years (s.d.)		14.62 (3.32)
Ethnicity, *n* (%)	Caucasian	404 (89.2)
	Black	11 (2.4)
	Indian/Pakistani/Bangladeshi	3 (0.7)
	Other	34 (7.5)
	Did not disclose	1 (0.2)
Relationship status, *n* (%)	Currently in long-term relationship	280 (61.8)
	Single, divorced, widowed	171 (37.7)
Mean number of lifetime traumatic events meeting DSM-IV criterion A (s.d.)		2.12 (1.96)
Mean number of traumatic events during training meeting criterion A (s.d.)		0.92 (1.89)
History of mental disorders, *n* (%)	Life-time prevalence of any disorder	188 (41.5)
	PTSD	68 (15.0)
	Social anxiety disorder	19 (4.1)
	Generalized anxiety disorder	11 (2.4)
	Major depressive episode	112 (24.7)
	Alcohol abuse	24 (5.2)
	Alcohol dependence	19 (4.1)
	Panic disorder	6 (1.3)
	Agoraphobia without panic disorder	6 (1.3)
	Substance abuse	14 (3.1)
	Substance dependence	12 (2.6)
	Obsessive–compulsive disorder	1 (0.22)
	Eating disorder	1 (0.22)
Psychological traits: mean (s.d.)	Dissociation	11.51 (6.59)
	Neuroticism	4.30 (3.14)
	Anxiety sensitivity	13.38 (8.72)
Cognitive risk factors: mean (s.d.)	Perceived resilience to stress	75.16 (10.63)
	Negative attitudes to emotional expression	10.38 (3.42)
	Depressive attributions	8.63 (7.26)
	Maladaptive post-traumatic cognitions	54.98 (26.73)
Cognitive responses to memories of stressful events: mean (s.d.)	Suppression	7.32 (3.85)
	Rumination	5.96 (4.80)
	Intentional numbing	2.65 (2.58)
Styles of coping with stress: mean (s.d.)	Substance use	2.70 (1.26)
	Behavioural disengagement	2.72 (1.05)
	Wishful thinking	7.32 (2.18)
Social support: mean (s.d.)	Social support (family and friends)	37.35 (6.41)
	Social support (work)	30.66 (4.72)

s.d., Standard deviation; DSM, Diagnostic and Statistical Manual of Mental Disorders, fourth edition; PTSD, post-traumatic stress disorder.

### Predictor variables

The following candidate predictors were selected on the basis of the available literature. All measures had established psychometric properties.

#### Psychiatric history

Trained psychologists administered the Structured Clinical Interview for the Diagnostic and Statistical Manual of Mental Disorders, fourth edition (DSM-IV) (SCID; First *et al.* 1996) to assess past and current mental disorders, excluding adult attention-deficit/hyperactivity disorder.

#### Trauma exposure

History of past traumatic events and trauma exposure during training were assessed with an extended version of the Life Events Checklist (LEC; Gray *et al.* 2004). For the purposes of the study, we added some traumas that paramedics may come across during the course of their training, such as ‘witnessing or coming across a suicide’ and ‘threatened or harassed by someone without a weapon’. For each trauma, participants also indicated whether they experienced fear, helplessness or horror. Pre-training trauma exposure was calculated as the total number of traumatic events meeting DSM-IV criterion A at baseline assessment. Participants also completed the extended LEC at each follow-up assessment. Trauma exposure during training was calculated as the total number of traumatic events meeting DSM-IV criterion A during the 2 years.

#### Dissociation

The brief Trait Dissociation Questionnaire (Murray *et al.* 2002) measured the frequency with which participants had a range of dissociative experiences such as depersonalization and derealization.

#### Neuroticism

Neuroticism was measured with a subscale of the Eysenck Personality Questionnaire (Eysenck & Eysenck, 1975).

#### Anxiety sensitivity

The Anxiety Sensitivity Inventory (Peterson & Reiss, 1992) asks participants to rate how fearful they are of specific anxiety-related sensations and to what extent they believe these sensations to have catastrophic consequences.

#### Perceived resilience to stress

The Connor–Davidson Resilience Questionnaire (CD-RISC; Connor & Davidson, 2003) assesses the extent to which participants endorse aspects of resilience such as confidence in their ability to deal with challenges and problems.

#### Attitudes to emotional expression

The Attitudes to Emotional Expression questionnaire (Williams *et al.* 1995) assesses negative attitudes towards talking about negative feelings or problems.

#### Depressive attributions

The Depressive Attributions Questionnaire (Kleim *et al.* 2011) assesses depressive attributional style, i.e. negative internal, stable and global attributions.

#### Maladaptive post-traumatic cognitions

The Posttraumatic Cognitions Inventory (Foa *et al.* 1999) assesses negative cognitions about the self or others after stressful events that have been shown to predict PTSD (Ehring *et al.* 2008). Participants answered the questions with respect to the most stressful event they had experienced.

#### Cognitive responses to memories of stressful events

The Responses to Intrusions Questionnaire (Clohessy & Ehlers, 1999; Ehring *et al.* 2008) assesses unhelpful responses to intrusive memories of stressful life events. The questionnaire has three subscales: Suppression, Rumination and Intentional Numbing.

#### Coping styles

Measures of unhelpful coping styles included the Substance Use and Behavioral Disengagement subscales from the Brief COPE Questionnaire (Carver *et al.* 1989), and a Wishful Thinking subscale from the Ways of Coping Scale that has shown to predict PTSD in emergency workers (Clohessy & Ehlers, 1999).

#### Social support

The Crisis Support Scale (Joseph, 1999) assessed perceived support from friends and family. Additional items measured perceived social support at work.

### Episodes of PTSD or MD during follow-up

At each 4-monthly follow-up assessment, participants completed the LEC to identify any stressful events. They were interviewed by trained psychologists over the telephone using the relevant SCID modules to determine whether they were currently experiencing an episode of PTSD (i.e. at least 1 month of the PTSD symptoms specified in DSM-IV with clinically significant distress or impairment in functioning) or MD (i.e. at least 2 weeks of the depression symptoms specified in DSM-IV with clinically significant distress or impairment in functioning), and if this was not the case, whether they had experienced an episode of PTSD or MD since the last assessment.

### Health outcomes at 2 years

#### Alcohol and drugs

Use of alcohol and street drugs was assessed with The Alcohol Use Disorders Identification Test (AUDIT; Babor *et al.* 2001).

#### Days off work

At 1 and 2 years, participants reported the total number of days off work due to illness and stress for the past year.

#### Weight changes

We asked participants to record fluctuations in weight (loss, gain, or no change) at 2-year follow-up.

#### Smoking

At 2-year follow-up, participants were asked by interview whether or not they were currently smokers, and whether they had been smokers at the beginning of training.

#### Burn-out

The Maslach Burnout Inventory – Human Services Survey (Maslach *et al.* 1996) assessed burn-out.

#### Insomnia

The Insomnia Severity Index (Morin *et al.* 2011) assessed the nature, severity, and impact of insomnia.

#### Quality of life

The Quality of Life Enjoyment and Satisfaction Questionnaire (Rapaport *et al.* 2005) assessed the participant's quality of life in 14 domains such as physical health, leisure activities and social relationships.

## Procedure

Participants were recruited and interviewed at the training centre when attending an intensive course during the first week of their paramedic training. If they consented to participate in the study, they attended the baseline interview that assessed current and past mental disorders the same week. They completed the self-report questionnaires to assess predictor variables prior to the interview.

Every 4 months, participants completed self-report questionnaires to assess trauma exposure and completed symptom measures. If they indicated they had been exposed to a stressful event, they were assessed by interview for PTSD and MD. At 12 and 24 months, all participants were interviewed with an extended interview to identify any further exposure to stressful events and PTSD or depression during the past year and whether or not they had received treatment. They also completed a brief set of self-report questionnaires assessing burn-out, days off work, weight, insomnia and quality of life.

## Data analyses

We used a hierarchical approach to data analysis. Point-biserial correlations were calculated to assess the associations between the 15 hypothesized predictors and episodes of PTSD and MD. Partial correlations were calculated between hypothesized predictors and outcome, controlling for psychiatric history and trauma exposure. Significant predictors identified in the partial correlation analyses for either disorder were entered into multiple logistic regression. All of these 12 variables showed zero-order correlations of *p* < 0.001 with at least one of the outcomes. Block 1 tested the effects of psychiatric history and trauma exposure, and block 2 tested whether the hypothesized predictors added to the prediction. Variables were centred for the analyses. Assessment of multicollinearity indicated that predictor inter-correlations and tolerance levels were within acceptable ranges. Odds ratios and 95% confidence intervals were calculated to provide a measure of effect size. Variables that violated assumptions of normality were square-root transformed.

## Results

### Episodes of PTSD or MD during 2 years of training

Of the 386 participants who provided follow-up data, 8.3% (*n* = 32) developed an episode of PTSD and 10.6% (*n* = 41) developed a major depressive episode at some time during follow-up, but in all but nine cases (2.3%) these reactions were short-lived episodes and had remitted by the next assessment 4 months later. The episodes were of moderate severity and were accompanied by moderate to severe clinical interference. Of the 32 participants who developed an episode of PTSD, 31.3% (*n* = 10) received treatment during follow-up, and 29.3% (*n* = 12) of the 41 participants who developed an episode of MD. Treatment ranged from counselling, peer support service, cognitive analytic therapy and cognitive–behaviour therapy (CBT) to medication. Of the nine participants who had recurrent PTSD or MD, 55.6% (*n* = 5) received treatment and failed to recover.

### Health outcomes at 2-year follow-up

As shown in Table 2, paramedics who developed an episode of PTSD or MD reported poorer well-being, as measured by more days off work, greater sleep problems and burn-out, and lower quality of life at the 2-year follow-up than those who did not develop these problems during training. Those with an episode of PTSD were also more likely to report weight gain over the 2 years than those without PTSD. For those who reported weight gain, the mean gain was 6.9 (s.d. = 4.5) kg. Participants who developed an episode of PTSD were more likely to be smokers at 2-year follow-up (40.7%) than those without PTSD (23.8%) [χ^2^ (1, *n* = 342) = 3.79, *p* = 0.05], whereas they reported no difference for baseline smoking [34.6% *v*. 27.3%, χ^2^ (1, *n* = 341) = 0.64, *p* = 0.42]. Smoking was unrelated to MD. Participants reported decreased use of alcohol at follow-up (mean AUDIT score at baseline: 6.35, s.d. = 4.21; at 2 years: 4.82, s.d. = 3.38, *F*_1,340_ = 23.95, *p* < 0.001) that was independent of PTSD or MD (all *p*'s > 0.227). At baseline, 6.8% of the participants reported occasional drug use (mainly cannabis), and 5% at 2 years. This reduction was found only for participants who did not develop PTSD or MD (*p* < 0.001).

**Table 2.** Health outcomes at 2 years for paramedics who developed episodes of PTSD or MD at some time during 2 years of training

	Episode of PTSD or MD during 2 years of training	
Outcome	Yes	None	Statistics
PTSD episode			
Mean time off work, days (s.d.)	10.70 (14.48)	6.30 (14.57)	*t*_381_ = 2.50, *p* = 0.013
Weight gain, *n*/*n* (%)	16/27 (59.3)	95/312 (30.4)	χ^2^_339_ = 9.37, *p* = 0.002
Sleep problems: mean (s.d.)	9.93 (5.79)	5.78 (4.88)	*t*_340_ = 4.18, *p* = 0.001
Burn-out: mean (s.d.)	44.25 (16.01)	36.57 (17.18)	*t*_340_ = 2.24, *p* = 0.026
Quality of life: mean (s.d.)	53.64 (14.11)	67.49 (14.74)	*t*_340_ = 4.70, *p* = 0.001
MD episode		
Mean time off work, days (s.d.)	14.70 (21.70)	5.7 (13.21)	*t*_44.64_ = 3.22, *p* = 0.002
Weight gain, *n*/*n* (%)	12/32 (37.5)	99/307 (32.2)	χ^2^_339_ = 0.36, *p* = 0.547
Sleep problems: mean (s.d.)	11.21 (6.01)	5.55 (4.65)	*t*_36.21_ = 5.25, *p* = 0.001
Burn-out: mean (s.d.)	43.73 (16.49)	36.48 (17.15)	*t*_340_ = 2.32, *p* = 0.021
Quality of life: mean (s.d.)	52.54 (14.99)	67.87 (14.41)	*t*_340_ = 5.79, *p* = 0.001

PTSD, Post-traumatic stress disorder; MD, major depression; s.d., standard deviation

### Predictors of episodes of PTSD and depression

Zero-order point-biserial correlations between baseline predictors and episodes of PTSD or MD during the 2-year follow-up are shown in Table 3. Demographic variables were unrelated to outcome. Most of the hypothesized predictors were associated with episodes of PTSD and MD. After controlling for psychiatric history and trauma exposure, the following groups of variables assessed at baseline continued to be associated with an episode of PTSD or MD during follow-up: psychological traits (dissociation, neuroticism), cognitive risk factors (low perceived resilience, depressive attributions, maladaptive post-traumatic cognitions), cognitive responses to memories of stressful events (suppression, rumination, intentional numbing), avoidant styles of coping with stress (behavioural disengagement, wishful thinking) and low social support.

**Table 3.** Point-biserial correlations between hypothesized predictors and episodes of PTSD or MD during 2 years of training, and partial correlations controlling for psychiatric and trauma history

	Episode of PTSD during 2 years of training		Episode of MD during 2 years of training	
Baseline measure	Zero-order point-biserial correlation (*n* = 386)	Partial correlation, controlling for psychiatric history and trauma exposure	Zero-order point-biserial correlation (*n* = 383)	Partial correlation, controlling for psychiatric history and trauma exposure
Psychiatric and trauma history				
History of mental disorders (SCID)	0.231***		0.233***	
Lifetime exposure to traumatic events at baseline^a^	0.057		0.127*	
Exposure to traumatic events from baseline to 2-year follow-up	0.032		0.113*	
Demographics				
Age	0.093	0.084	0.093	0.075
Gender	0.066	0.067	0.044	0.080
Ethnic minority	0.018	0.026	−0.011	−0.007
Years of full-time education	−0.012	−0.008	0.036	0.048
In long-term relationship	−0.100	−0.087	−0.033	−0.024
Psychological traits				
Trait dissociation	0.185***	0.130*	0.216***	0.164**
Neuroticism	0.202***	0.139**	0.241***	0.189***
Anxiety sensitivity	0.146**	0.091	0.141**	0.092
Cognitive risk factors				
Perceived resilience to stress	−0.153**	−0.120*	−0.227***	−0.210***
Negative attitudes towards emotional expression	0.105*	0.076	0.103*	0.066
Depressive attributions	0.162**	0.103*	0.216***	0.163**
Maladaptive post-traumatic cognitions^a^	0.219***	0.156**	0.233***	0.165**
Cognitive responses to stressful memories				
Suppression	0.176***	0.125*	0.098	0.049
Rumination	0.243***	0.186***	0.241***	0.180***
Intentional numbing	0.203***	0.139**	0.192***	0.122*
Styles of coping with stress				
Substance use^a^	0.011	−0.035	0.135**	0.091
Behavioural disengagement	0.163**	0.128*	0.190***	0.153**
Wishful thinking	0.119*	0.066	0.219***	0.186***
Social support				
Perceived social support (family and friends)	−0.198***	−0.154**	−0.235***	−0.182***
Perceived social support (work)	−0.154**	−0.123*	−0.193***	−0.164**

PTSD, Post-traumatic stress disorder; MD, major depression; SCID, Structured Clinical Interview for Diagnostic and Statistical Manual of Mental Disorders, fourth edition.

a Variable was square-root transformed.

* *p* < 0.05, ** *p* < 0.01, *** *p* < 0.001.

Multiple logistic regression analyses identified unique predictors of episodes of PTSD (Table 4). In block 1 past psychiatric history, lifetime exposure to traumatic events and trauma exposure during training significantly predicted PTSD [χ^2^ (3, *n* = 386) = 21.26, *p* = 0.001, Nagelkerke *R*^2^ = 0.123], and history of mental disorders predicted unique variance. The prediction of PTSD episodes was improved when the hypothesized predictors identified in the partial correlation analyses were entered in block 2 [χ^2^ (15, *n* = 386) = 43.64, *p* = 0.001, Nagelkerke *R*^2^ = 0.246]. Rumination in response to unwanted memories of stressful events uniquely predicted an episode of PTSD during follow-up, as did a history of mental disorders.

**Table 4.** Results of multiple logistic regressions: regression coefficients, Wald statistics, ORs with 95% CIs for predictors of episodes of PTSD and MD

	Episode of PTSD during 2 years				Episode of MD during 2 years			
	*B* (s.e.)	Wald	*p*	OR (95% CI)	*B* (s.e.)	Wald	*p*	OR (95% CI)
Block 1: psychiatric history and trauma exposure only								
History of mental disorders	1.81 (0.45)	16.23	0.001	6.09 (2.53–14.67)	1.54 (0.38)	16.50	0.001	4.66 (2.22–9.79)
Lifetime trauma exposure at baseline^a^	0.04 (0.33)	0.02	0.90	1.04 (0.54–2.0)	0.34 (0.30)	1.31	0.25	1.40 (0.79–2.50)
Trauma exposure during 2-year follow-up	0.02 (0.02)	0.50	0.48	1.02 (0.97–1.06)	0.04 (0.02)	3.70	<0.05	1.04 (0.99–1.01)
Block 2: all predictors								
Psychiatric history and trauma exposure								
History of mental disorders	1.27 (0.50)	6.48	0.01	3.55 (1.34–9.39)	0.84 (0.44)	3.63	0.06	2.31 (0.98–5.44)
Lifetime trauma exposure at baseline^a^	−0.05 (0.38)	0.02	0.89	0.95 (0.45–1.99)	0.50 (0.34)	2.25	0.13	1.65 (0.86–3.19)
Trauma exposure during 2-year follow-up	0.01 (0.03)	0.26	0.61	1.01 (0.96–1.07)	0.05 (0.02)	4.42	0.04	1.05 (1.00–1.10)
Psychological traits								
Dissociation	0.02 (0.04)	0.15	0.70	1.02 (0.94–1.10)	0.02 (0.04)	0.38	0.54	1.02 (0.95–1.10)
Neuroticism	0.12 (0.09)	1.76	0.19	1.12 (0.95–1.33)	0.12 (0.08)	2.43	0.12	1.13 (0.97–1.32)
Cognitive risk factors								
Perceived resilience to stress	−0.21 (0.02)	0.79	0.36	0.98 (0.93–1.03)	−0.04 (0.02)	3.80	<0.05	0.96 (0.92–1.00)
Depressive attributions	−0.07 (0.04)	2.86	0.09	0.93 (0.86–1.01)	−0.07 (0.04)	2.96	0.09	0.94 (0.87–1.01)
Maladaptive post-traumatic cognitions^a^	−0.01 (0.15)	0.01	0.92	0.99 (0.73–1.33)	−0.08 (0.14)	0.28	0.58	0.93 (0.70–1.23)
Cognitive responses to stressful memories								
Suppression	0.08 (0.07)	1.50	0.22	1.08 (0.95–1.23)	−0.01 (0.06)	0.05	0.82	0.99 (0.88–1.11)
Rumination	0.11 (0.05)	4.17	0.04	1.11 (1.00–1.23)	0.09 (0.05)	3.09	0.08	1.09 (0.00–1.19)
Intentional numbing	−0.52 (0.09)	0.31	0.58	0.95 (0.79–1.14)	−0.05 (0.09)	0.24	0.63	0.96 (0.80–1.15)
Styles of coping with stress								
Behavioural disengagement	0.33 (0.23)	2.03	0.15	1.38 (0.89–2.16)	0.15 (0.21)	0.48	0.49	1.16 (0.77–1.74)
Wishful thinking	−0.09 (0.12)	0.66	0.42	0.91 (0.73–1.14)	0.17 (0.11)	2.42	0.12	1.18 (0.96–1.47)
Social support								
Social support – general	−0.04 (0.04)	1.34	0.25	0.96 (0.89–1.03)	−0.04 (0.04)	1.08	0.30	0.96 (0.89–1.04)
Social support – work	−0.03 (0.04)	0.41	0.52	0.97 (0.88–1.07)	−0.03 (0.05)	0.57	0.45	0.97 (0.88–1.06)

OR, Odds ratio; CI, confidence interval; PTSD, post-traumatic stress disorder; MD, major depression; s.e., standard error.

a Variable was square-root transformed.

For episodes of MD, block 1 (past psychiatric history and trauma exposure) significantly predicted episodes of MD [χ^2^ (3, *n* = 383) = 27.18, *p* = 0.001, Nagelkerke *R*^2^ = 0.139]. The prediction significantly improved with the addition of the psychological variables entered in block 2 [χ^2^ (15, *n* = 383) = 58.39, *p* = 0.001, Nagelkerke *R*^2^ = 0.287]. Low perceived resilience at baseline and degree of exposure to traumatic events during training uniquely predicted the risk of having an episode of MD during follow-up.

## Discussion

Overall, the present cohort of newly recruited paramedics coped well with the demands of training. Although nearly all of them experienced a potentially traumatic event during the 2-year study period, the majority did not develop episodes of PTSD or MD, and those who did usually recovered within a few months. However, the significant minority of 8.3% who experienced an episode of PTSD, and 10.6% who experienced an episode of MD suffered clinically significant distress and interference with functioning. At 2-year follow-up these participants reported more days off work, poorer sleep, greater burn-out, lower quality of life and greater weight gain (PTSD only), indicating that episodes of PTSD and MD are predictive of long-term poorer well-being and potentially physical health.

The results support the need for adequate support for emergency workers who are exposed to potentially traumatic events and, similar to studies following emergency workers who responded to the World Trade Center attacks (i.e. Pietrzak *et al.* 2014), underscore the need for prevention, screening and treatment efforts that target high-risk workers. Those at risk of developing PTSD or MD may benefit from specific training programmes to boost their resilience. This study showed that participants at risk of developing an episode of PTSD or depression could be identified within the first week of their paramedic training programme. In line with previous research (Wittchen *et al.* 2013), a history of mental disorders predicted episodes of PTSD and depression during training, leading to a six-fold (PTSD) and five-fold (MD) increase in risk. The role of degree of trauma exposure differed between episodes of PTSD and MD. Participants’ risk of developing an episode of PTSD during training was neither correlated with the total number of adverse events experienced before training or during training, consistent with theories and other data showing that the individual's reaction to trauma is more predictive than trauma exposure *per se* (Declerq *et al.* 2011). In contrast, degree of trauma exposure was associated with the risk of developing MD, suggesting a cumulative risk of different exposures that is consistent with previous findings (Kendler *et al.* 1999).

Since past psychiatric history and trauma exposure cannot be modified, future resilience interventions for emergency workers are best targeting modifiable predictors. Predictors of episodes of PTSD and depression identified in this study were consistent with cognitive models, highlighting the role of maladaptive cognitive risk factors such as attributional style and maladaptive post-traumatic cognitions, cognitive responses to memories of stressful events, avoidant coping, psychological traits such as a tendency to dissociative reactions and neuroticism, and low social support.

The multiple logistic regression analysis showed that while the predictors shared substantial common variance, rumination about memories of stressful events prior to starting paramedic training uniquely predicted an episode of PTSD during the 2-year study period. These results are consistent with a number of studies that have demonstrated support for the link between rumination and the development and maintenance of PTSD (e.g. Clohessy & Ehlers, 1999; Laposa & Alden, 2003; Kleim *et al.* 2007). Rumination is a core intervention target in cognitive therapy for PTSD (e.g. Ehlers *et al.* 2003) and it remains to be tested whether therapeutic procedures used to modify rumination could be used to foster resilience.

For episodes of MD, low levels of psychological resilience predicted unique variance, consistent with the literature (O'Rourke *et al.* 2010). Whilst our findings link low levels of resilience to developing depression, Metcalfe (1968) found that the tendency to ruminate and lack of adaptability (i.e. low levels of resilience) were personality features of people who developed depression even once they recovered, highlighting the link between resilience and depression almost 50 years ago. It would seem that a possible target for emergency staff training programmes could be resilience training, i.e. training to increase confidence in dealing with stressful events at work.

Preliminary research suggests that resilience can be trained. For example, Steinhardt & Dolbier (2008) showed that students experiencing increased academic stress reported greater resilience, better coping and fewer symptoms of depression after resilience training (psychoeducation and CBT techniques such as modifying interpretations of stressful events) than a wait-list control. Watkins *et al.* (2009) demonstrated that it is possible to modify rumination. Participants with dysphoria who ruminated were allocated to training in concrete thinking about events, bogus training or a wait list. Participants who received concreteness training experienced greater decreases in rumination than those who received no training. Finally, trauma-focused cognitive therapy for PTSD targets rumination as a core process that maintains PTSD. Patients are taught how to spot when they are ruminating and how to transform their ruminative thoughts to present-focused thinking. Randomized controlled trials demonstrate significant reductions in PTSD symptoms and rumination in response to unwanted memories (e.g. Ehlers *et al.* 2014).

The incidence of episodes of PTSD and depression in the present sample is in line with previous work of European samples regularly exposed to trauma (Wittchen *et al.* 2013). Of our sample, 41.5% had a psychiatric history prior to training similar to findings from the US National Comorbidity Survey, which identified nearly 50% of respondents as meeting criteria for at least one lifetime psychiatric disorder. The rates of past MD and past PTSD at the start of training were slightly higher in our sample compared with rates in the general population but consistent with overall rates of anxiety disorder (23.9%) and mood disorders (19.3%; Kessler *et al.* 1994). It would seem that our paramedic sample has experienced past PTSD at a greater rate than the general population, which may be a factor that draws them to emergency work. More research is needed to determine why trainee paramedics have high rates of past PTSD over other psychiatric disorders. Our sample had low rates of social anxiety disorder (SAD) at the start of training and this may reflect the likelihood that people with SAD are unlikely to be drawn to paramedic training, possibly because this profession involves daily exposure to strangers.

The results of the negative effects of episodes of PTSD and depression on health outcomes at the 2-year follow-up extend existing research. Losses of work productivity linked to common mental disorders are comparable with those of physical illnesses (Smit *et al.* 2006). Burn-out may increase risk of future episodes of depression (Ahola *et al.* 2006) and affects organizations and individuals through high staff turnover, absenteeism, and a decrease in the amount and quality of care provided by staff to service users. The weight gain observed in those who developed PTSD replicates previous findings (Talbot *et al.* 2013) and is an established risk factor for cardiovascular disease and other health-related outcomes. Sleep problems have also been linked to negative health outcomes, including diabetes and weight gain (Knutson *et al.* 2007) and increased risk for cardiovascular disease (Mullington *et al.* 2009). Depression increases the risk of cardiovascular disease due to associated changes in nervous system activation, cardiac rhythm disturbances, systemic and localized inflammation, and hypercoagulability that negatively influence the cardiovascular system (Joynt *et al.* 2003). PTSD similarly increases risk for cardiovascular disease (Kubzansky *et al.* 2007). Thus, even when participants recovered from PTSD and MD, they may retain risk for physical disease through chronic poor sleep and weight gain.

Our study has several limitations. We assessed paramedics every 4 months. Monitoring symptoms with such frequency may influence the natural course of development of psychopathology since monitoring offers the opportunity to reflect on one's distress and make changes, such as seeking support, if needed. However, monitoring of PTSD symptoms has shown limited success in reducing PTSD symptoms (Ehlers *et al.* 2003). We recorded days absent from work by self-report without verification with managers. This was necessary to maintain confidentiality but may represent an underestimation of the number of days our participants were absent from work. While we assessed alcohol, drug and tobacco use at baseline and 2-year follow-up, we unfortunately did not assess daily exercise or caffeine consumption, which could influence well-being and health and should be assessed in future research. We did not assess participants’ weight at baseline and relied on them to record gain in weight by 2-year follow-up, as such we cannot comment on the percentage increase in body weight. We relied on the LEC to record exposure to traumatic events during the course of the study. The LEC is unable to assess repeated exposure to the same trauma. It is thus conceivable that participants may have experienced more than one event of a particular trauma type during a 4-month assessment period, which may have led to an underestimation of the total number of traumatic events they experienced. However, the interviews did not suggest that this was the case. Participants may have under-reported their reactions to traumatic events and failed to record that they responded with fear, helplessness or horror, which could also lead to an underestimation of the number of traumatic events experienced.

Our sample consisted of newly recruited paramedics and it remains unclear whether the findings generalize to other emergency workers, military personnel or to the general population. However, our sample was assessed at the start of their paramedic training when they were more likely to share similarities rather than differences with the other populations. Nonetheless, this is the first large-scale prospective study of paramedics assessed prior to repeated exposure to trauma and, as such, offers a unique contribution to the field of prediction and prevention. The predictors identified in this study could serve as targets to modify in future resilience programmes, which may help to avert the development of PTSD and depression in at-risk individuals and the linked risk for physical disease.
